# Mapping Small Extracellular Vesicle Secretion Potential in Healthy Human Gingiva Using Spatial Transcriptomics

**DOI:** 10.3390/cimb47040256

**Published:** 2025-04-07

**Authors:** Blanka Maria Borowiec, Małgorzata Blatkiewicz, Marta Dyszkiewicz-Konwińska, Dorota Bukowska, Bartosz Kempisty, Marcin Ruciński, Michał Nowicki, Joanna Budna-Tukan

**Affiliations:** 1 Department of Histology and Embryology, Poznan University of Medical Sciences, 60-781 Poznań, Poland; blanka.borowiec@student.ump.edu.pl (B.M.B.); mblatkiewicz@ump.edu.pl (M.B.); marcinruc@ump.edu.pl (M.R.); mnowicki@ump.edu.pl (M.N.); 2 Doctoral School, Poznan University of Medical Sciences, 60-812 Poznań, Poland; 3 Department of Diagnostics, Poznan University of Medical Sciences, 60-812 Poznań, Poland; m.dyszkiewicz@ump.edu.pl; 4 Department of Preventive Dentistry, Collegium Medicum in Bydgoszcz, Faculty of Medicine, Nicolaus Copernicus University in Torun, 85-067 Bydgoszcz, Poland; 5 Department of Diagnostics and Clinical Sciences, Institute of Veterinary Medicine, Nicolaus Copernicus University in Torun, 87-100 Toruń, Poland; dbukowska@umk.pl; 6 Division of Anatomy, Department of Human Morphology and Embryology, Faculty of Medicine, Wroclaw Medical University, 50-368 Wrocław, Poland; bartosz.kempisty@umw.edu.pl; 7 Prestage Department of Poultry Science, College of Agriculture and Life Sciences, North Carolina State University, Raleigh, NC 27695-7608, USA; 8 Department of Veterinary Surgery, Institute of Veterinary Medicine, Nicolaus Copernicus University, 87-100 Toruń, Poland; 9 Center of Assisted Reproduction, Department of Obstetrics and Gynecology, University Hospital and Masaryk University, 625 00 Brno, Czech Republic; 10 Department of Immunology, Poznan University of Medical Sciences, 60-806 Poznań, Poland; 11 Department of Anatomy and Histology, Collegium Medicum, University of Zielona Gora, 65-046 Zielona Góra, Poland

**Keywords:** spatial transcriptomics, oral mucosa, gingiva, small extracellular vesicles, regeneration

## Abstract

Regenerative processes occur at various levels in all organisms, yet their complexity continues to raise new questions about their mechanisms. It has been demonstrated that small extracellular vesicles (sEVs), secreted by all cells and influencing their function, play a significant role in regeneration. In the context of regenerative processes, oral mucosal tissues consistently receive interest, as they are among the most rapidly healing tissues in the human body. In this study, we utilized spatial transcriptomics to map gene expression to specific spatial locations within the gingiva tissue section, using publicly available transcriptomic data. This analysis revealed new insights into this tissue and the biogenesis of sEVs within it. The identified clusters encompassed two main regions—the epithelium and lamina propria—as well as minor niches within them. Using Gene Ontology (GO) analysis, we identified two clusters most enriched in extracellular vesicle-related GO processes. These included the superficial and deeper layers of the sulcular epithelium, one of the most peripheral regions of the gingiva. Of the 43 genes identified in the literature as having a potential or documented role in sEVs biogenesis, 12 were selected for further analysis. *MUC1*, *SDCBP2*, and *VPS37B* showed clear specificity and the highest expression in the superficial layer of the sulcular epithelium. *CHMP4C* also exhibited high expression in this layer, though its levels were comparable to the outer layer of the oral epithelium. Other well-established sEVs marker genes, such as *ANXA2*, *CD9*, *CD63*, *CD81*, *FLOT1*, *RAB22A*, *RAB27B*, and *RAB5A*, were also expressed in the examined tissue; however, their expression was not specifically exclusive to the sulcular epithelium. Our study is the first to perform a meta-analysis of available gingival transcriptomic data in the specific context of sEVs biogenesis. The presented data and conclusions provide new insights into the role of different structures within healthy human gingiva and shed new light on both known and potential markers of sEVs biogenesis. These findings may contribute to the development of regeneration-targeted research, especially on oral tissues.

## 1. Introduction

One fundamental characteristic of living organisms is their susceptibility to various forms of damage, ranging from macroscopic to molecular scales. While the nature and extent of such damage depend on numerous factors, it is typically followed by the activation of regenerative processes [1]. These processes are greatly complex, requiring numerous stages, structures, and pathways, contributing to their diversity [2]. This variability is evident not only across different species but also within single organisms, where distinct tissues exhibit unique regenerative capacities [3].

In humans, this principle holds true. Tissues such as embryonic and fetal structures, the liver, and the intestinal epithelium demonstrate particularly efficient regenerative capabilities, with embryonic and fetal tissues being especially notable [4]. Interestingly, specific adult tissues have been reported to exhibit regenerative processes resembling the highly efficient mechanisms observed in fetal development, even in fully mature individuals [5].

A tissue that demonstrates exceptional regenerative properties is the oral mucosa. Based on function, this tissue can be categorized into three types: (1) masticatory mucosa, comprising the free and attached gingiva and the hard palate; (2) lining mucosa, encompassing the cheek, vestibule, inner lips, and lateral surfaces of the alveolar process, soft palate, ventral tongue surface, and floor of the mouth; and (3) specialized mucosa, limited to the dorsum of the tongue [6]. The oral mucosa usually consists of three distinct layers: the oral epithelium, the lamina propria, and the submucosa; however, it varies [7]. Its layered structure is sometimes compared to that of the skin, yet its regenerative capacity surpasses that of the largest human organ in many respects.

Although the mechanisms underlying rapid and highly effective healing with minimal scarring remain the subject of ongoing research, the literature identifies several contributing factors, including the tissue’s rich vascularization, the presence of saliva, persistent low-level inflammation, and a dense microbiome [8]. Counterintuitively, the latter two factors, often perceived as detrimental to healing, play key roles in this process. A mild and controlled inflammation state keeps the tissue “ready” to rapidly initiate repair processes [7]. At the same time, the dense microbiome regulates the overgrowth of invading bacteria by competing for resources and adhesion sites, additionally balancing pro- and anti-inflammatory processes through, e.g., the induction of regulatory T cells [9].

This state of readiness was first termed homeostatic inflammation in 1976 by Page and Schroeder, who provided a detailed classification of periodontal lesions induced by inflammation [10]. Among their classifications, the term “initial lesion” was used to describe the state of a gingiva within 2–4 days after plaque accumulation, characterized by acute inflammation, including vasculitis and increased neutrophil migration into the junctional epithelium and gingival sulcus [10]. The subsequent stages—early, established, and advanced lesions—progressively increased in severity, involving processes such as, among others, lymphocyte infiltration, the onset of basal cell proliferation in the junctional epithelium, connective tissue destruction, and the potential apical migration of the junctional epithelium, indicating early pocket formation, ultimately leading to pocket formation and alveolar bone and periodontal ligament loss [10].

Slightly more than 20 years later, Kinane and Lindhe proposed a revised classification, noting that the previous framework was based primarily on animal models and tissue samples from human adolescents, which did not fully reflect the situation in human adults [11,12]. Their updated classification introduced several changes, the most notable being the distinction of a new stage, pristine gingiva, characterized by histological perfection without a trace of inflammatory infiltrate. Additionally, the original definition of the “initial lesion” was reinterpreted, not as a pathological condition, but as a normal, healthy gingival state [11,12]. According to this classification, the initial lesion was defined by the following, respectively: (1) the vasculitis of vessels beneath the junctional epithelium, (2) the exudation of fluid into tissues and the gingival sulcus, (3) the increased migration of leukocytes into the junctional epithelium and gingival sulcus, (4) the presence of serum proteins, particularly fibrin, in extravascular spaces, (5) alteration in the most coronal portion of the junctional epithelium, and (6) the loss of perivascular collagen [11,12].

The next stage, early gingivitis, remained classified according to the previous system as the “early lesion” and was characterized by the following, respectively: (1) the accentuation of the features described for the initial lesion, (2) the accumulation of lymphoid cells immediately below the junctional epithelium, (3) cytopathic alterations in resident fibroblasts, (4) the further loss of the collagen fiber network in the marginal gingiva, and (5) the early proliferation of the basal cells of the junctional epithelium [11,12].

This classification reshaped the understanding of inflammation in gingival tissue, further deepening insights into its strong regenerative potential. This enabled a significant expansion of the list of factors that, when considered, may contribute to maintaining this potential at a remarkably high level.

In addition to the previously mentioned factors, another noteworthy element is small extracellular vesicles (sEVs), which, similarly to inflammation and the dense microbiome, may also exhibit a dual nature. According to the latest MISEV (Minimal Information for Studies of Extracellular Vesicles) guidelines, sEVs are extracellular vesicles smaller than 200 nm [13]. These membrane-bound vesicles, released by all eukaryotic and prokaryotic cells, consist of a protein-rich lipid bilayer and carry diverse cargo, including proteins, RNAs, mRNAs, miRNAs, lipids, and metabolites [14]. This diversity enables them to participate in critical processes such as intercellular communication, immunoregulation, and tissue regeneration [15].

As research into regenerative processes expands, growing evidence highlights the medical potential of sEVs, with promising experimental findings laying the groundwork for future clinical applications [16]. While the entire oral mucosa is exposed to damage and pathogens, the gums are particularly notable due to their structure, which facilitates bacterial accumulation and growth, leading to, e.g., gingivitis [17]. Additionally, they are prone to minor, routine injuries, such as those caused by inadequate daily dental hygiene, e.g., harsh flossing or brushing [18]. This makes the gingiva an ideal focus for inflammation and cancer research. Evidence suggests that sEVs derived from gingival mesenchymal stem cells (GMSCs) can influence macrophage polarization and phenotype under periodontitis-related inflammatory conditions [19]. According to the findings, macrophages play a key role in defending against periodontitis-causing pathogens, with the ability to polarize into pro-inflammatory (M1) or anti-inflammatory (M2) phenotypes. GMSC-derived sEVs were shown to inhibit M1 activation and promote M2 polarization, thus emphasizing their agency [19]. Additionally, in periodontitis, sEVs from TNF-α-conditioned GMSCs were found to promote M2 macrophage polarization by increasing CD73 expression [20]. The local administration of these sEVs in mice reduced periodontal bone resorption and osteoclast numbers, demonstrating their double role in modulating inflammation and inhibiting osteoclastogenesis [20].

To better understand regeneration mechanisms, it is essential to use tools that analyze both relationships between cells and their unique properties. Spatial transcriptomics, a technique first introduced about 30 years ago, has recently gained significant attention for its ability to address this need [21]. It enables gene expression analysis within cells’ natural tissue environment, combining transcriptomic data with spatial positioning to create a detailed and informative profile [22]. This approach provides diverse insights, including cell localization within preserved tissue architecture, tissue composition, and interactions between cells, cell clusters, or even whole compartments [23].

Our study leverages spatial transcriptomics to investigate extracellular vesicles, explicitly focusing on sEVs in human gingival tissue. Through an extensive literature review, we gathered together and identified potential and already confirmed genetic markers linked to sEVs and analyzed their expression using publicly available spatial transcriptomic data from healthy gingival tissue [24]. This approach allowed us to pinpoint tissue regions with the highest potential for sEV secretion by mapping gene expression at precise spatial locations. By uncovering the sEV secretion potential of individual cells and areas, our findings offer new insights that refine the research focus and enable the generation of highly targeted and relevant data.

## 2. Materials and Methods

Publicly available spatial transcriptomic data of the oral mucosa were retrieved from the Gene Expression Omnibus (GEO) (accession GSE206621). All original data-obtaining processes are described in detail by Caetano et al. [24].

### 2.1. Data Acquisition and Preprocessing

The raw Visium output files (including the barcode list, features, count matrix, and tissue positions) were downloaded and examined. Spots corresponding to tissue-associated regions were retained by filtering tissue_positions_list.csv to include only entries with a tissue indicator value of 1. Barcodes not present in the filtered file were discarded, ensuring that subsequent analyses focused solely on valid tissue-associated spots.

### 2.2. Seurat Object Creation

The filtered count data were loaded into R (v4.1.2; R Core Team 2021) and used to construct a Seurat object, specifying the “Spatial” assay [25,26]. Genes detected in at least 1% of the retained spots were kept, and all others were excluded to reduce sparsity. A Visium tissue image was then linked to the Seurat object by subsetting the image data to include only the filtered barcodes corresponding to the tissue-associated spots.

### 2.3. Data Normalization and Quality Control

All analyses were performed using R (v4.1.2; R Core Team 2021, available at https://cran.r-project.org, accessed on 2 February 2025) with Seurat (v5.2.1) and related packages. The total gene counts per spot were visualized in violin plots to assess data quality. Normalization was conducted using log-normalization procedures to obtain comparable expression values across spots. Genes with the highest variability were identified using the default methods in Seurat [26].

### 2.4. Dimensional Reduction and Clustering

Principal component analysis (PCA) was carried out on the scaled data. A shared nearest-neighbor (SNN) graph was then created, and clustering was performed at multiple resolutions. Uniform Manifold Approximation and Projection (UMAP) was employed to visualize the high-dimensional data in two-dimensional space, enabling clear delineation of tissue heterogeneity and identification of spatially localized clusters.

### 2.5. Differential Expression Analysis

Marker gene detection for each cluster was conducted using likelihood-ratio tests for single-cell gene expression within the Seurat framework. Only positive markers with an adjusted *p*-value < 0.05 and average log2 fold change > 1 were retained as significantly upregulated. Heatmaps were generated to depict the top marker genes across clusters, offering insights into transcriptional signatures and potential functional roles in the tissue context. This approach follows previous work by Blatkiewicz et al. [27], which demonstrated the utility of heatmap visualization in capturing transcriptional signatures within tissue contexts.

### 2.6. Gene Ontology Analysis

Gene Ontology enrichment was carried out using SeuratExtend (available at https://github.com/huayc09/SeuratExtend, accessed on 2 February 2025) to identify pathways associated with the cellular component category. Genes enriched in specific terms were visualized with spatial feature plots and violin plots to illustrate differential expression patterns across clusters. This approach facilitated a deeper understanding of subpopulation-specific transcriptomic functions within the oral mucosa samples.

### 2.7. Additional Data Visualization

Advanced plots, including multi-marker feature plots and bar plots of cluster distributions, were generated to elucidate the cell populations’ compositional and spatial diversity. Spatial dimension plots, along with standard dimensional reduction plots, aided in confirming cluster identity and exploring potential biological relevance.

### 2.8. Software and Hardware

All steps were executed in R on the Windows 10 operating system. The key R packages included Seurat [26], SeuratExtend (v 1.1.3 available at https://github.com/huayc09/SeuratExtend, accessed on 2 February 2025), ggplot2 (v 3.5.1) [28], patchwork (v 1.3.0) [29], openxlsx (4.2.8) [30], Matrix (v 1.7.3) [31], and R.utils (v 2.13) [32]. Statistical tests, including Wilcoxon rank-sum tests for comparisons across clusters [33], were conducted where relevant.

## 3. Results

**Figure 1:**
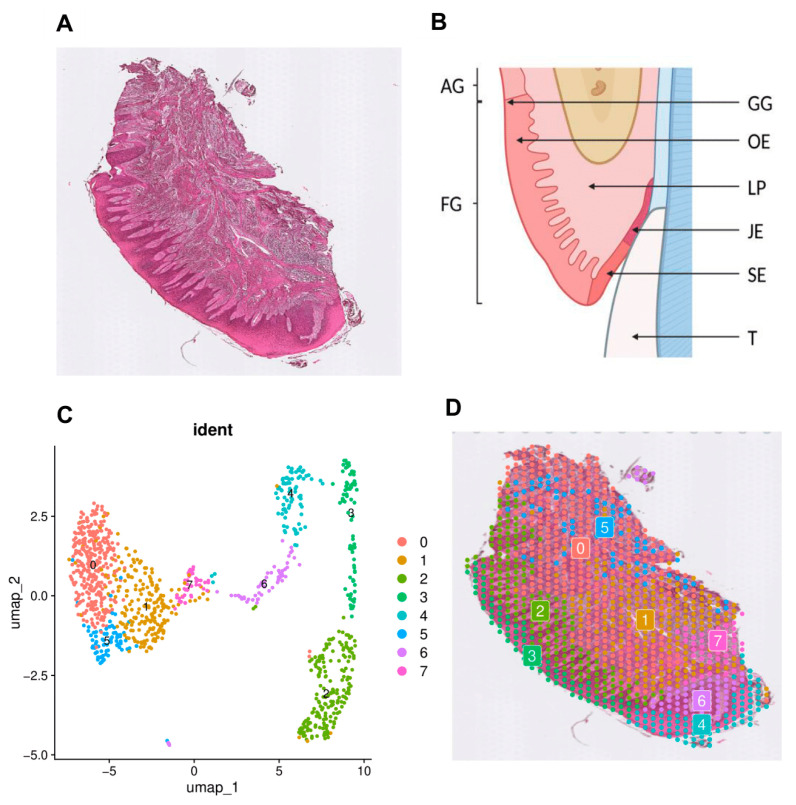
The transcriptomic landscape of a healthy mucosa section—the gingiva of an adult human. (**A**) A histological image of the healthy oral mucosa section stained with hematoxylin and eosin (H&E). (**B**) A schematic illustration of the gingival area: AG—attached gingiva; FG—free gingiva; GG—gingival groove; OE—oral epithelium; LP—lamina propria; JE—junctional epithelium; SE—sulcular epithelium; T—tooth. (**C**) A Uniform Manifold Approximation and Projection (UMAP) plot of transcriptomic clusters identified in the tissue. A specific color distinguishes each cluster and corresponds to a distinct transcriptionally unique cell population or region of the mucosa: 0—stromal and stromal reticular cells (lamina propria); 1—stromal immune cells (lamina propria); 2—basal epithelial and stromal subepithelial cells (oral epithelium and lamina propria cusp); 3—suprabasal epithelial cells (oral epithelium); 4—suprabasal epithelial cells (sulcular epithelium); 5—stromal endothelial and immune cells (lamina propria); 6—basal epithelial and stromal subepithelial cells (sulcular epithelium and lamina propria cusp); 7—stromal immune cells (lamina propria). (**D**) Clusters from the UMAP representation are depicted as color-coded dots in the histological image of the oral mucosa slide. The colored grids correspond to spatially resolved transcriptomic spots, with each color representing a specific cluster from the UMAP analysis. Figure 1B was created with BioRender.com.

Meta-analysis of the spatial transcriptomics landscape was performed based on publicly available data (GSE206621) within a healthy mucosal tissue section [24]. Spatial transcriptomics provided a high-resolution map of gene expression heterogeneity across the mucosa by integrating histological features with molecular profiling. This process demonstrated the complexity of tissue organization at both the cellular and subcellular levels, highlighting key functional niches. The histological image of the section, stained with hematoxylin and eosin (H&E), provided an anatomical context for spatial gene expression profiling (Figure 1A). The characteristic stratified squamous epithelium was visible, forming a protective barrier against the oral cavity, while the underlying connective tissue layer (lamina propria) supported the local vasculature and immune cell populations. This histological overview confirmed the tissue’s integrity and the absence of pathological changes, serving as a baseline for spatial transcriptomic profiling. Additionally, the main elements of the gingival area are presented in a schematic in Figure 1B. Utilizing the spatial transcriptomics data, eight distinct transcriptional clusters (labeled 0–7) were identified, each corresponding to a specific gene expression signature visualized through a UMAP plot (Figure 1C) and mapped onto the histological section (Figure 1D). Mapping the histological features onto the spatial transcriptomics clusters revealed an alignment between the tissue’s anatomical domains and the molecular signatures of clusters 0 to 7. Figure 1 was created using a publicly available tissue section provided alongside the transcriptomic dataset by Caetano et al. (Gene Expression Omnibus, accession number: GSE206621). Analyses of this tissue section conducted by the same research team are detailed in the publication “Spatially resolved transcriptomics reveals pro-inflammatory fibroblasts involved in lymphocyte recruitment through CXCL8 and CXCL10.” [24]. The standalone image of the tissue section is also accessible under the same accession number (GSE206621).

### 3.1. Description of Assigned Clusters

As a result of the conducted analyses, it was possible to identify eight clusters within the tissue section, indicating distinctive regions.

The epithelium, characterized by its multiple cellular layers from the basal to superficial, primarily corresponds to clusters 2, 3, 4, and 6. The more externally placed cluster 3 indicates the external region of the keratinized oral epithelium consisting of suprabasal epithelial cells, while cluster 2 indicates a deeper papillary region, which contains basal epithelial and stromal subepithelial cells. Cluster 4 indicates the more external region of the non-keratinized region of the sulcular epithelium, while cluster 6 indicates the deeper papillary region, which also contains basal epithelial and stromal subepithelial cells. The basal layer, composed of columnar to cuboidal cells with uniform nuclei, anchors firmly to the basement membrane and supports the dynamic turnover of cells [34], reflected in the proliferative and structural genes detected in this cluster. Due to their location, subepithelial stromal cells are in constant crosstalk with the epithelium [35]; they clearly differ from deeper stromal cells, as seen in the demarcated clustering. The junctional epithelium, which would usually be in a neighborhood of clusters 4 and 6, is not included in this particular section of the gingiva. However, the potential influence of this cluster on the closely located clusters, like clusters 4 and 6, should be acknowledged.

The lamina propria, situated beneath the epithelium, consists of a collagen-rich matrix interspersed with fibroblasts, small vessels, and immune cells [36], exhibiting a close correlation with cluster 0, consisting of stromal and stromal reticular cells with a probable predominance of fibroblasts. This cluster demonstrates the heightened expression of genes associated with extracellular matrix remodeling and stromal support, thereby aligning with its anatomical distribution within the connective tissue. Additionally, clusters 1, 5, and 7 map to regions encompassing immune and vascular elements within the lamina propria and adjacent areas. Cluster 1 consists mainly of stromal immune cells with a probable predominance of B lymphocytes and plasma cells. Similar to this cluster, cluster 7 is composed mainly of stromal immune cells, likely dominated by B and T lymphocytes, as well as NK cells. In contrast, cluster 5 primarily contains stromal endothelial cells and fibroblasts, along with a noticeable number of immune cells. The gene signatures in these regions suggest their potential roles in immune surveillance, vascular function, and tissue homeostasis, which aligns with the observed presence of immune cells and small blood vessels. Additionally, Figure 1B presents a schematic illustration of the gingiva with detailed annotations of its regions.

In summary, the molecular clustering directly reflects the anatomical and functional segmentation: the epithelium (clusters 2, 3, 4 and 6), the collagenous, stromal compartment (lamina propria) (cluster 0), and the vascular-immune niches within the lamina propria (clusters 1, 5 and 7). This integrated view underscores how distinct cellular subpopulations and tissue structures in the healthy buccal gingival margin cooperate to maintain overall tissue integrity.

### 3.2. Description of Differentially Expressed Genes in Assigned Clusters

**Figure 2:**
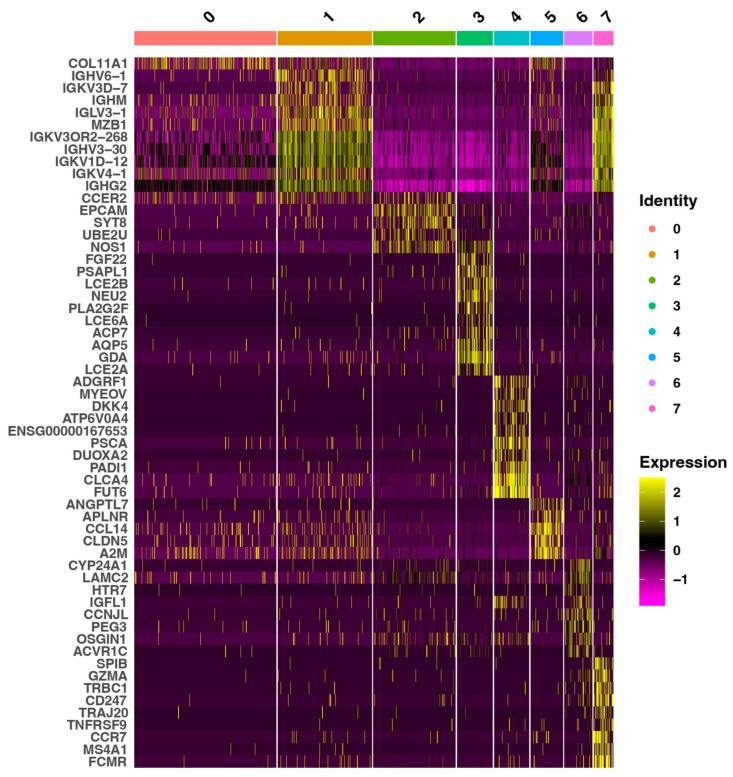
A heatmap of differentially expressed genes across eight identified transcriptional clusters. Each cluster’s top expressed genes (z-score normalized) are visualized on the heat map. Each column represents one characterized cluster, whereas each row corresponds to a single gene. The color scale transitions from purple (low expression) through black (median expression) to yellow (high expression). The numbers indicate the following clusters: 0—stromal and stromal reticular cells (lamina propria); 1—stromal immune cells (lamina propria); 2—basal epithelial and stromal subepithelial cells (oral epithelium and lamina propria cusp); 3—suprabasal epithelial cells (oral epithelium); 4—suprabasal epithelial cells (sulcular epithelium); 5—stromal endothelial and immune cells (lamina propria); 6—basal epithelial and stromal subepithelial cells (sulcular epithelium and lamina propria cusp); 7—stromal immune cells (lamina propria).

Next, we created a heatmap with the top expressed genes to illustrate the differentially expressed genes (DEG) across all characterized transcriptional clusters (Figure 2).

#### 3.2.1. Description of DEGs in Cluster 0

The meta-analysis revealed that for cluster 0 (stromal and stromal reticular cells in lamina propria), the most expressed gene is *COL11A1* (Collagen Type XI Alpha 1 Chain), which is an extracellular matrix (ECM) component, linked to stromal support, aligning with the anatomical distribution of collagen fibers within the lamina propria. Although not well characterized, the *CCER2* (Coiled-Coil Glutamate Rich Protein 2) gene is also upregulated in cluster 0. This suggests the presence of cells with high metabolic activity, such as fibroblasts. It may also indicate the activity of stromal endothelial cells, which support vascular permeability and immune cell migration.

#### 3.2.2. Description of DEGs in Cluster 2

Subsequently, cluster 2 (oral epithelium and lamina propria cusp containing basal epithelial and stromal subepithelial cells) reveals the identification of *EPCAM* (Epithelial Cell Adhesion Molecule), a hallmark of epithelial lineage, as well as *SYT8* (Synaptotagmin 8) and *NOS1* (Nitric Oxide Synthase 1), which are typically featured in neurotransmission or secretory pathways. Furthermore, the high expression of *UBE2U* (Ubiquitin-Conjugating Enzyme E2 U) in cluster 2 highlights metabolic and regulatory demands within this population. The expression pattern of these genes in cluster 2 indicates the presence of a unique niche within the buccal gingival margin.

#### 3.2.3. Description of DEGs in Cluster 3

The suprabasal epithelial layer, designated as cluster 3, is distinguished by a population of epithelial cells that exhibit multiple functional facets. Among these is *FGF22* (Fibroblast Growth Factor 22), which has been implicated in tissue remodeling [37]. Other notable genes include *LCE2A*, *LCE2B*, and *LCE6A* (Late Cornified Envelopes 2A, 2B, 6A), which are essential for terminal differentiation and the barrier function of stratified epithelia, and *PLA2G2F* (Phospholipase A1 group IIF), which contributes to inflammatory or defense responses. Additionally, *AQP5* (Aquaporin 5), a gene encoding a water channel protein that facilitates water transport across cell membranes, plays a key role in regulating water balance in the oral mucosal epithelium [38], further confirming the accurate identification of this cluster.

#### 3.2.4. Description of DEGs in Cluster 4

Meanwhile, cluster 4 (suprabasal epithelial cells in sulcular epithelium) is represented by overexpression of mostly *CLCA4* (Chloride Channel Accessory 4) and *ATP6V0A4* (ATPase H+-Transporting V0 Subunit A4) genes, which are related to support ion transport and pH regulation. These genes potentially help to maintain a microenvironment conducive to epithelial and immune cell function. As an epithelial tissue, it also serves as a barrier. It may be evidenced by the higher expression of *DUOXA2* (Dual Oxidase Maturation Factor 2), which is involved in the production of hydrogen peroxide (H_2_O_2_) in epithelial cells, a compound associated with antibacterial and oxidative functions [39]. This may indicate the role of this area in protection against bacterial infections, which, due to its location and direct proximity to a tooth, is particularly important given its constant exposure to bacterial biofilm containing resident or transient microorganisms. *FUT6* (Fucosyltransferase 6), which is also highly expressed and involved in synthesizing glycoconjugates on the surface of epithelial cells, may also play a role in the response to close and constant contact with dental plaque pathogens [40]. This suggests its potential function in defense mechanisms by modulating the bacterial microbiota and limiting pathogen adhesion.

#### 3.2.5. Description of DEGs in Cluster 5

In cluster 5 (stromal endothelial and immune cells in lamina propria), several genes associated with vascular and immune-modulatory functions are overexpressed, predominantly *ANGPTL7* (Angiopoietin Like 7), an angiogenic factor involved in ECM organization and homeostasis. Furthermore, the observed upregulation of *APLNR* (Apelin Receptor) suggests a potential role in angiogenesis and blood pressure regulation. In addition, *CCL14* (C-C Motif Chemokine Ligand 14) is implicated in immune cell recruitment and inflammatory responses [41], while *A2M* (Alpha-2-Macroglobulin) is involved in the modulation of inflammation and tissue repair [42]. Collectively, these observations imply the potential involvement of these genes in angiogenesis, vascular integrity, and immune cell recruitment.

#### 3.2.6. Description of DEGs in Cluster 6

The overexpression of genes in cluster 6 (basal epithelial and stromal subepithelial cells (sulcular epithelium and lamina propria cusp)) suggests that this subset of cells may be involved in dynamic epithelial remodeling, the stress response, and signaling interactions that help maintain the integrity and function of the tissue. Especially, the overexpression of *PEG3* (Paternally Expressed 3) and *OSGIN1* (Oxidative Stress Induced Growth Inhibitor 1) highlight cellular stress responses and growth regulation, while *IGFL1* (Insulin Growth Factor-Like Family Member 1 ) and *CCNJL* (Cyclin J Like) point to proliferative or growth factor-related pathways that may support tissue renewal. The high expression of *LAMC2* (Laminin Subunit Gamma 2), a gene encoding a component of laminin that plays a key role in the adhesion of epithelial cells to the basement membrane and extracellular matrix [43], is noteworthy. This is reasonable given that cluster 6, despite forming the sulcular epithelium together with cluster 4, is located closer to the lamina propria than cluster 4. Additionally, the elevated expression of *CYP24A1* (Cytochrome P450 Family 24 Subfamily A Member 1) might be an important aspect. It is involved in regulating vitamin D metabolism in tissues and may influence calcium-phosphate balance and the inflammatory response. Furthermore, it modulates cell differentiation and the course of inflammation [44]. The above observations suggest that cluster 6 may serve various roles, including regulating epithelial attachment to connective tissue, epithelial regeneration and proliferation, and even the response to mechanical and oxidative stress.

#### 3.2.7. Description of DEGs in Clusters 1 and 7

Meanwhile, clusters 1 and 7 (stromal immune cells) have some visible similarities. The expression of the immunoglobulin genes *IGHM* (Immunoglobulin Heavy Constant Mu), *IGHG2* (Immunoglobulin Heavy Constant Gamma 2), and *IGHA1* (Immunoglobulin Heavy Constant Alpha 1) is observed in these clusters, suggesting the presence of active B cell or plasma cell populations. These findings suggest a potential role in local immune surveillance, corroborating the hypothesis that immune cells are present even in healthy mucosal tissues and protect against microbial challenges in the oral cavity [45]. Additionally, the increased expression of genes such as *IGHV3-30* and *IGHV6-1* (Immunoglobulin Heavy Variables 3-30 and 6-1) may indicate active recombination and antibody diversity targeting antigens associated with periodontal bacteria, which include not only transient bacteria but also bacteria resident in the oral cavity. As mentioned above, contact with these bacteria may only cause the state known as the ‘initial lesion’ and not necessarily the ‘early lesion’. This is suggested by the fact that these genes encode the variable region of the immunoglobulin heavy chain, determining antibody specificity for particular antigens. Moreover, *MZB1* (Marginal Zone B and B1 Cell-Specific Protein 1) also plays a role in B cell maturation, supporting antibody production [46]. Furthermore, cluster 7 is rich in genes with both T and B lymphocyte activity, suggesting their role in immune survey and adaptive immunity. The overexpression of *GZMA* (Granzyme A), *TNFRSF9* (TNF Receptor Superfamily Member 9), and *CD247* (CD247 Molecule) are hallmark T cell markers linked to cytotoxic function and co-stimulation. At the same time, *SPIB* (Spi-B Transcription Factor) and *MS4A1* (Membrane Spanning 4-Domains A1) are related to B cell development and maturation. Additionally, the upregulation of *TRAJ20* (T Cell Receptor Alpha Joining 20) may suggest that this cluster consists of active T cells engaged in the immune response.

The observed indications of involvement in immunological processes occurring in clusters 4 and 6, particularly in cluster 6, may be attributed to their proximity to the predominantly immunological cluster 7. These observations may not only indicate the general role of cluster 7 in immune responses but can suggest that this area begins to represent criteria linked to the “early lesion”, given the possible presence of the above-mentioned active lymphocytes.

Together, these cluster-specific expression patterns complement the spatial maps of the healthy mucosa. The heatmap highlights cellular and functional heterogeneity underlying oral mucosa homeostasis by revealing distinct transcriptomic signatures for epithelial, stromal, and immune cell compartments.

### 3.3. Description of Gene Ontology Processes

**Figure 3:**
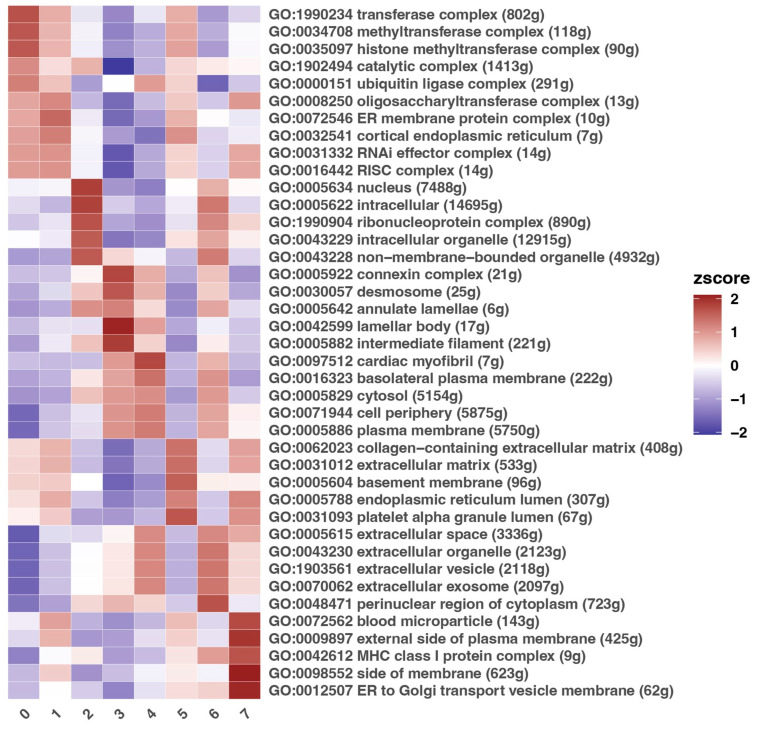
A heatmap of the Gene Ontology (GO) term enrichment scores (z-scores) for each cluster. Rows correspond to GO terms linked to cellular components, while columns indicate individual clusters. Red shading (z-score closer to +2) signifies relative enrichment, whereas blue shading (z-score closer to −2) indicates relative depletion. The numbers indicate the following clusters: 0—stromal and stromal reticular cells (lamina propria); 1—stromal immune cells (lamina propria); 2—basal epithelial and stromal subepithelial cells (oral epithelium and lamina propria cusp); 3—suprabasal epithelial cells (oral epithelium); 4—suprabasal epithelial cells (sulcular epithelium); 5—stromal endothelial and immune cells (lamina propria); 6—basal epithelial and stromal subepithelial cells (sulcular epithelium and lamina propria cusp); 7—stromal immune cells (lamina propria).

To interpret the biological functions and processes associated with differentially expressed genes in each cluster, Gene Ontology (GO) term analysis was performed for eight transcriptomic clusters (0–7) (Figure 3). The heatmap represents the z-score values of enriched GO terms associated with cellular components, providing insight into the functional roles of different cell populations within the mucosal tissue.

Notably, clusters 0, 1, and 5 exhibit significant similarities in the enrichment of terms related to the complex formation and regulation of cellular processes and intracellular localization, suggesting an involvement of these clusters in coordinating fundamental cellular processes. Moreover, clusters 2 and 3 are enriched in GO terms related to intracellular complexes and ribosomal functions, implying that these clusters may represent immune cells or cells with high protein synthesis and intracellular trafficking activity.

Furthermore, clusters 4 and 6 also present some similarities with the terms associated with their potential involvement in intracellular processes, reflecting their adaptation in intracellular communications (GO, 0005829, GO: 0071944). Moreover, clusters 4 and 6 show a pattern that suggests that these cells are active producers of vesicle-mediated communication, which is connected to the extracellular vesicle biogenesis (GO:1903561, GO:007062). The same clusters are also enriched in processes related to the extracellular space and its components (GO:0005615, GO:0043230), indicating a connection to the site of secretion and activity of extracellular vesicles, among other elements. Their elevated scores for the extracellular vesicles-associated GO term imply that these epithelial, basal epithelial, and stromal subepithelial cells may actively secrete sEVs or micromolecules that regulate immune cells or by facilitating ECM remodeling and tissue repair processes. These findings indicate that clusters 4 and 6 may harness extracellular vesicles and sEVs as key mediators of cell–cell interactions in maintaining and regulating mucosal homeostasis. Meanwhile, clusters 5, 6, and 7 have overlapping enrichment for immune- or vesicle-related functions and may encompass different immune or immunomodulatory cell populations.

In summary, the observed enrichment patterns reveal the diverse roles of distinct cell populations within the mucosa.

### 3.4. Description of Selected Genes with a Potential or Documented Role in sEV Biogenesis

In the subsequent stage of this study, a detailed analysis was performed of the potential or already confirmed markers linked to sEV biogenesis and their transcriptional landscape in the healthy human mucosa. A literature review identified a range of sEV markers (Table A1). Each marker was then subjected to a more detailed analysis regarding its documented or potential role in sEV biogenesis. Based on this analysis, 43 markers were selected: *ALIX*, *ANXA2*, *ANXA5*, *ANXA11*, *BAG6*, *CD9*, *CD63*, *CD81*, *CD82*, *CERK*, *CLTC*, *CHMP1A*, *CHMP2A*, *CHMP4B*, *CHMP4C*, *FLOT1*, *HSPA8*, *HSP90AA1*, *ICAM1*, *ITGB3*, *LGALS3BP*, *MFGE8*, *MUC1*, *PLD2*, *PTEN*, *RAB5A*, *RAB7A*, *RAB11A*, *RAB11B*, *RAB22A*, *RAB27A*, *RAB27B*, *RAB35*, *SDCBP2*, *SMPD3*, *SNAP23*, *STX4*, *TP53*, *TSG101*, *VAMP7*, *VPS4A*, *VPS4B*, and *VPS37B*.

Not all genes were detected in the transcriptome of the analyzed tissue fragment. Among the database containing the transcriptome of the analyzed tissue sample, 12 of the genes mentioned above were identified: *ANXA2*, *CD9*, *CD63*, *CD81*, *CHMP4C*, *FLOT1*, *MUC1*, *RAB5A*, *RAB22A*, *RAB27B*, *SDCBP2*, and *VPS37B* (Table 1). Subsequently, the expression levels and distribution across clusters were examined for each of the selected genes.

#### 3.4.1. Description of Clusters 4 and 6 in Context of Selected Genes

**Figure 4:**
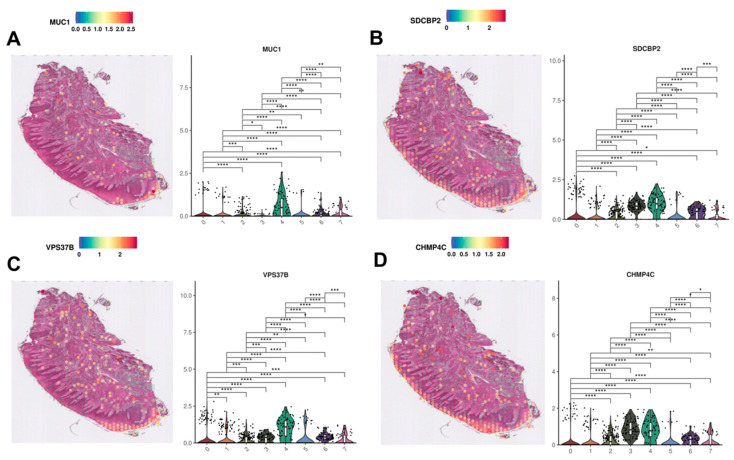
The spatial distribution and cluster expression of genes associated with extracellular vesicle biogenesis in the healthy human mucosa (**A**–**D**). Each part of the selected gene includes an H&E-stained tissue section overlaid with color-coded spots reflecting gene expression (scale shown at the top of the graph). Warmer hues (orange/red) indicate higher transcript abundance. Furthermore, violin plots illustrate expression levels in clusters 0–7. * *p* < 0.05; ** *p* < 0.01; *** *p* < 0.001; **** *p* < 0.0001. The numbers indicate the following clusters: 0—stromal and stromal reticular cells (lamina propria); 1—stromal immune cells (lamina propria); 2—basal epithelial and stromal subepithelial cells (oral epithelium and lamina propria cusp); 3—suprabasal epithelial cells (oral epithelium); 4—suprabasal epithelial cells (sulcular epithelium); 5—stromal endothelial and immune cells (lamina propria); 6—basal epithelial and stromal subepithelial cells (sulcular epithelium and lamina propria cusp); 7—stromal immune cells (lamina propria).

Since GO enrichment analysis revealed that clusters 4 and 6 present the highest potential in this area (Figure 3), a more in-depth investigation was conducted in order to identify potential markers and confirm the presence of already known sEVs markers. The analysis revealed that the most overexpressed genes in clusters 4 and 6 are *MUC1* (Mucin 1), *VPS37B* (VPS37B Subunit Of ESCRT-I), *SDCBP2* (Syndecan Binding Protein 2), *and CHMP4C* (Charged Multivesicular Body Protein 4C), while all of them are predominantly expressed only in cluster 4 (Figure 4A–D). However, the expression of *CHMP4C* demonstrates high expression not only in cluster 4 but also in cluster 3. The highest expression in the sulcular suprabasal epithelium is most prominently observed for the genes *MUC1* and *VPS37B*. For *SDCBP2*, the plot indicates dominance in cluster 4; however, it is also noticeably expressed in cluster 3. A similar pattern of a difference between clusters 3 and 4 is observed for *CHMP4C*.

#### 3.4.2. Description of Selected Genes That Were Not Exclusively Characteristic of Clusters 4 and 6

Not all markers of biogenesis identified in Table 1 exhibit distinct expression in clusters 4 and 6. Many display comparable or higher expression across multiple clusters. While the previously described genes (Figure 4) are the primary focus of this study, it is also necessary to analyze the remaining markers, which could enhance the understanding of the nature of the clusters of interest.

**Figure 5:**
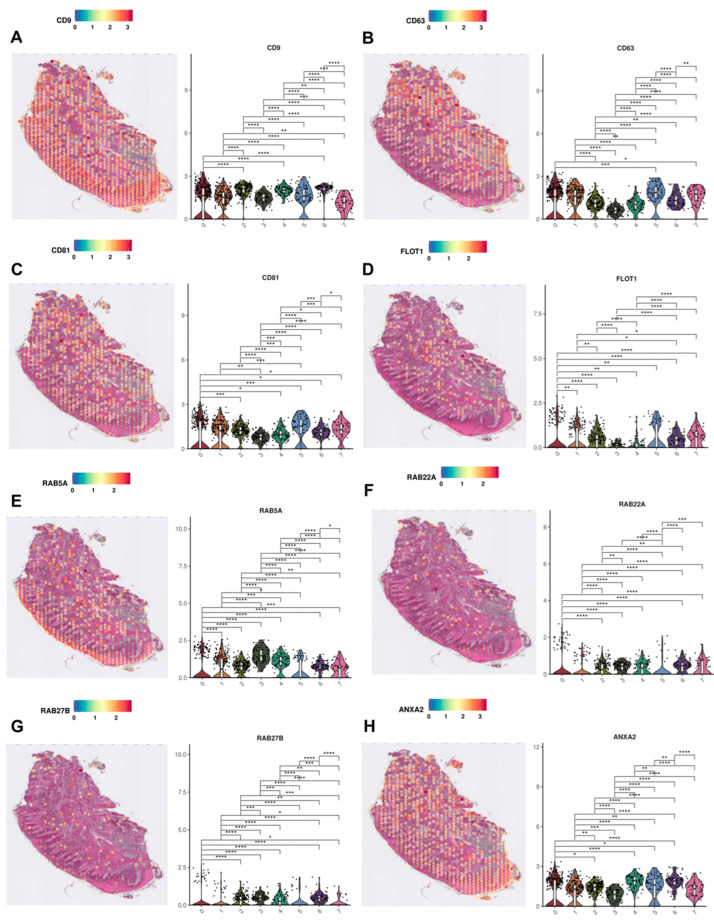
The spatial distribution and cluster expression of genes associated with extracellular vesicle biogenesis in the healthy human mucosa (**A**–**H**). Each part of the selected gene includes an H&E-stained tissue section overlaid with color-coded spots reflecting gene expression (scale shown at the top of the graph). Warmer hues (orange/red) indicate higher transcript abundance. Furthermore, violin plots illustrate expression levels in clusters 0–7. * *p* < 0.05; ** *p* < 0.01; *** *p* < 0.001; **** *p* < 0.0001. The numbers indicate the following clusters: 0—stromal and stromal reticular cells (lamina propria); 1—stromal immune cells (lamina propria); 2—basal epithelial and stromal subepithelial cells (oral epithelium and lamina propria cusp); 3—suprabasal epithelial cells (oral epithelium); 4—suprabasal epithelial cells (sulcular epithelium); 5—stromal endothelial and immune cells (lamina propria); 6—basal epithelial and stromal subepithelial cells (sulcular epithelium and lamina propria cusp); 7—stromal immune cells (lamina propria).

GO process analysis identified clusters 4 and 6 as representative of the superficial and deeper layers of the sulcular epithelium. The analyzed section also includes the second epithelium (oral epithelium), represented by clusters 2 and 3. As anticipated, some markers exhibit similar expression in both epithelium types (Figure 5). *CD9*, *CD63*, and *CD81 (CD9*, *CD63* and *CD81 Molecules)* are among the most recognized markers, not only for sEV biogenesis but for sEVs in general. Of these, only *CD9* demonstrates significantly elevated expression in clusters encompassing both epithelia’s superficial and deeper layers, with the highest expression in cluster 4. Within the epithelium-related clusters (2, 3, 4 and 6), the highest *CD9*, *CD63*, and *CD81* expression is observed in cluster 2. *FLOT1* (Flotillin 1), another well-documented sEV marker, exhibits the highest expression in cluster 2, which represents a deeper layer of the oral epithelium. Among other popular sEV markers, *RAB5A*, *RAB22A*, and *RAB27B* (RAB5A, RAB22A, RAB27B, Members RAS Oncogene Family) also show elevated expression in the epithelium-related clusters. For *RAB5A*, the superficial layers of both epithelia stand out, with cluster 3 surpassing cluster 4 in expression levels. For *RAB22A*, the overall expression is lower; however, clusters 2, 4, and 6 stand out in the epithelium-related clusters, with the highest expression observed in cluster 2. Similarly, for *RAB27B*, the overall expression is also lower and it stands out in the same clusters 2, 4, and 6; however, the highest expression is found in cluster 6. In the epithelium-related clusters, *RAB27B* shows higher expression in clusters associated with deeper epithelial layers, with the highest expression observed in cluster 6. The gene *ANXA2* (Annexin II) exhibits elevated expression across more than half of the presented clusters. In the epithelium-related clusters, clusters 4 and 6 surpass clusters 2 and 3, with the highest expression observed in cluster 6.

Although the differences in the expression of individual genes between clusters are not as apparent as in Figure 4, it is still possible to identify the clusters in which the selected genes show the highest expression. Within the epithelium-related clusters, cluster 2 shows the highest *CD63*, *CD81*, *FLOT1*, and *RAB22A* expression. Cluster 6 exhibits the highest expression of *ANXA2* and *RAB27B*, while clusters 3 and 4 are characterized by elevated levels of *RAB5A* and *CD9*, respectively. These findings indicate that within the analyzed genes, most of them demonstrate the highest expression in cluster 2. The second in order is cluster 6. Both clusters correspond to the deeper layers of the oral and sulcular epithelia, respectively. An interesting finding is that the most commonly used sEV markers, such as tetraspanins and Rab-family proteins, generally do not exhibit the highest expression in the outer layers of either epithelium. Given the characteristics of these regions, their constant exposure to pathogens, and frequent microdamage, they would be expected to be highly involved in sEV biogenesis. This may be a remarkable insight, suggesting that in gingival tissue, vesicles identified using these markers are more likely to originate from deeper tissue layers rather than the epithelium.

### 3.5. Summary of the Results

In summary, the results of the in-depth spatial transcriptomic meta-analysis of a healthy gingival tissue section using publicly available data (GSE206621, [21]) provided important insights. By mapping gene expression heterogeneity in the epithelium and lamina propria layers, we identified eight distinct clusters, each corresponding to specific regions of the analyzed tissue section. GO process analysis revealed two clusters with particularly enriched processes related to various aspects of sEV biogenesis. Genes with a documented or potential role in sEV biogenesis were mapped onto the tissue section. Subsequently, we characterized genes with the most distinct expression in key clusters 4 and 6 (representing the superficial and deeper layers of the sulcular epithelium) nominated by GO analysis. Additionally, we described genes that exhibited less distinct expression but have been previously confirmed in the literature to be involved in sEV biogenesis.

Our findings indicate that *MUC1*, *VPS37B*, and *SDCBP2* show the most distinctive expression in clusters 4 and 6 despite not being well-established markers of sEV biogenesis. This study establishes a reference for gingival spatial transcriptomics, providing new insights into sEV biogenesis in the sulcular epithelium.

## 4. Discussion

Research on regeneration processes and their related elements has long been, and continues to be, a subject of interest for numerous research groups and academic institutions. To fully understand their mechanisms, consequences, and roles within tissues of interest, it is essential to examine the smaller structural units that comprise these tissues, as well as their complex interactions. Spatial transcriptomics is still not a widely adopted method. Consequently, analyses of oral cavity tissues using this approach are not extensively documented, with most studies being published only within the past 2 years. Existing analyses focus on tissues such as the palate, tongue, cheek, and gingiva, often emphasizing cancer research [24,35,56,57,58,59]. However, none of these studies focus mainly on sEV-related topics.

Using publicly available spatial transcriptomics data, our meta-analysis represents the first attempt to identify gene expression patterns associated with sEV biogenesis in the gingiva.

The above meta-analysis was conducted using publicly available transcriptomic data obtained from a healthy human gingival tissue sample [24]. The analysis identified two primary histological layers: the epithelium and the lamina propria. Within these layers, eight distinct cell clusters (0–7) were identified and represented areas containing suprabasal and basal epithelial cells along with stromal subepithelial cells for both oral and sulcular mucosa, stromal, stromal reticular, and stromal endothelial cells of the lamina propria, along with immune cells of the lamina propria.

This study aimed to assess the spatial distribution of potential or already confirmed markers of sEV biogenesis in regions of the gingiva. To achieve this, a Gene Ontology (GO) biological process analysis was conducted, which identified two clusters as being the most enriched in processes critical to this study’s objectives: GO:1903561—extracellular vesicles; GO:007062—extracellular exosome (sEV); GO:0005615—extracellular space; and GO:0043230—extracellular organelles. These clusters, designated as 4 and 6, correspond to the outer and deeper layers of the sulcular epithelium, specifically the suprabasal epithelial cells (cluster 4) and the basal epithelial and stromal subepithelial cells (cluster 6).

A thorough literature review identified 43 genes with either a documented or potential role in the biogenesis of sEVs. Of these, 14 genes exhibit expression in the analyzed gingival tissue section. In the most relevant clusters for this study, clusters 4 and 6, the genes *MUC1*, *VPS37B*, *SDCBP2*, and *CHMP4C* are distinguished within cluster 4 (Figure 4). Genes such as *ANXA2*, *CD9*, *CD63*, *CD81*, *FLOT1*, *RAB5A*, *RAB22A*, and *RAB27B* also demonstrate significant expression levels, this time within all epithelium-related clusters (2, 3, 4 and 6). However, they are not as specific, displaying similar expression levels in clusters associated with the other type of epithelium: the oral epithelium (Figure 5). Analyzing the genes most specific to clusters chosen in the GO analysis (Figure 4) revealed that cluster 4 predominates. This suggests that cluster 4, which consists of suprabasal epithelial cells of the sulcular epithelium, has the highest potential for enhanced sEV biogenesis and genes such as *MUC1*, *VPS37B*, and *SDCBP2* can serve as potential markers of sEV biogenesis in the sulcular epithelium.

Among anatomical sites, the oral cavity is one of the most exposed to external factors. In terms of surface area, it is surpassed by the skin; however, unlike the skin, which is not an internal part of the body, the interaction between the external and internal environments in the oral cavity is more direct. The oral cavity is in constant contact with bacteria, both from its own microbiota and external strains, as well as viruses, fungi [60], harmful substances such as tobacco [61], irritating spicy or acidic foods [62], and mechanical damage caused, among other factors, by improper oral hygiene [18]. It should be noted that periodontal bacteria may include both resident and transient species [63]. Whether resident bacteria contribute to a pathological state depends on various factors, including disruptions in oral homeostasis [64]. Nevertheless, the presence of both types of bacteria consistently elicits certain types of responses from the oral mucosa [65].

Although it is not explicitly stated, it can be assumed that the oral mucosa has developed some of the fastest and most efficient wound healing processes due to its constant and intense exposure to harmful factors. Among the most exposed components of the oral cavity is the gingiva; its structure and location contribute to its high level of susceptibility [66]. The presence of dental plaque, which consists of bacteria and can persist or accumulate with inadequate oral hygiene, particularly exposes two parts of the gingiva to pathogens: the junctional epithelium (JE) and the sulcular epithelium (SE). The JE is located deepest and closest to the tooth, attaching the connective tissues to the tooth surface [67]. Positioned slightly above it, the SE lines the gingival sulcus. The oral epithelium (OE), which covers the outer surface of the gingiva, is also frequently exposed to pathogens; however, it does not experience constant exposure to the same extent as the junctional and sulcular epithelia.

In this study, examining the expression of selected genes in JE was impossible due to its absence in the analyzed tissue section. It is important to emphasize that for a comprehensive understanding, the inclusion of JE would be necessary, given that one of the key factors contributing to efficient tissue regeneration is a high cell turnover rate. In JE, this rate is 4–6 days [68], making it one of the highest among solid tissues in the human body, alongside the colonic epithelium (3–5 days) [69]. However, it should be noted that the cell turnover rate in the SE is also remarkably high (6–12 days) [68] and that its region was identified in GO analyses as exhibiting the highest intensity of extracellular vesicle-related processes. While these findings suggest the need to consider both epithelia in future studies, they also provide deeper insights into structures that have previously been classified as secondary in terms of regenerative potential within the gingival tissue.

The cluster most closely positioned in the region of missing JE is cluster 7. Although it was not identified in the GO analysis as one of the clusters most enriched in sEV-related processes, the expression of genes associated with sEV biogenesis is frequently elevated within it. Cluster 7 was classified as highly associated with immune cells and, consequently, with immunological processes. It is necessary to remember that, according to the current classification, the healthy gingiva is described as an “initial lesion” which exhibits particular traits of inflammation. Despite the tissue being healthy, it cannot be fully ruled that the area in the vicinity of the deeper parts of JE begin to form an “early lesion”, according to the classification by Kinane and Lindhe. This hypothesis could be supported by the distinctive appearance of the tissue observed in this sample region, possibly indicating lymphocytic infiltration. This hypothesis is further supported by the high expression levels of *IGHG2* (Immunoglobulin Heavy Constant Gamma 2) and *IGKV* (Immunoglobulin Kappa Variable Cluster), which may indicate an activity of B cells and active immunoglobulin synthesis. This may suggest an ongoing immune response targeted at combating pathogens.

The closest counterpart to the absent JE in the analyzed tissue section is the sulcular epithelium, which is fully preserved. The sulcular epithelium surrounds the most apical part of the gingiva and is also in constant or close contact with the tooth [70]. In clusters 4 and 6, which encompass this region, we observed varying expression levels of genes associated with sEV biogenesis. The elevated and distinct expression of the *MUC1*, *SDCBP2*, and *VPS37B* genes provides significant insights. These findings suggest that the sulcular epithelium, particularly its outer layer, might play a crucial barrier role, contribute to immune responses, and actively interact with bacteria (either resident and/or transient) present in the oral cavity, especially near the tooth.

The *MUC1* gene encodes a membrane-bound glycoprotein belonging to the mucin family, which serves as a key component of the mucus covering epithelial surfaces, including the oral cavity [71]. Although it is not a solid structure, it provides a form of chemical and, to some extent, physical barrier that protects tissues from pathogens and, to some extent, minimizes mechanical damage [72,73]. While its main function is to form a mucus barrier that protects the epithelium—primarily by preventing bacterial adhesion—it can also act as a receptor for certain bacteria [74,75,76]. This dual role allows for the appropriate modulation of the immune response. There is evidence of increased *MUC1* expression following bacterial contact. The study suggests that bacterial presence likely triggers phosphorylation-dependent signaling, which may influence *MUC1* expression levels [76]. Additionally, the presence of microbes and the products secreted by them also leads to increased *MUC1* expression [77]. The highest expression levels observed in the outermost part of the sulcular epithelium suggest that this region experiences the greatest exposure to bacterial biofilm (containing either resident and/or transient bacteria) from dental plaque, which could confirm the above-mentioned findings.

Beyond the above-described functions, *MUC1* plays an important role in cellular signaling, contributing to the transmission of signals related to environmental stress responses [50,78]. For example, it undergoes phosphorylation by tyrosine kinases such as EGFR (Epidermal Growth Factor Receptor), which activates signaling pathways like NF-κB, primarily involved in bacterial infection responses, and the MAPK/ERK pathway, which regulates cell proliferation under stress conditions [50]. This signaling activity appears particularly important given the frequent exposure of the sulcular epithelium to pathogens.

In the context of sEV biogenesis, the cytoplasmic domain of the MUC1 protein, MUC1-C, plays a vital role. MUC1-C directly interacts with RAB27A, a GTPase essential for sEV docking and secretion into the extracellular space [50]. Studies have shown that MUC1-C inhibition reduces sEV secretion from cancer cells, particularly RKO (colorectal cancer cells) and BT-594 (triple-negative breast cancer cells). The decreased expression of key sEV markers, such as CD9, CD63, and CD81, further confirms this. These findings suggest a correlation between high MUC1-C expression and increased sEV secretion [50]. Although these studies have been conducted in cancer cells exhibiting high *MUC1* expression, non-cancerous cells in contact with bacteria also show the increased expression of this gene, as mentioned above. Therefore, these findings may extend beyond cancer cells.

SDCBP2 also known as Syntenin-2, is the closest homolog protein of the more broadly studied Syntenin-1, encoded by the *SDCBP* (Syndecan Binding Protein) gene [79]. They are both scaffolding proteins that link syndecans (a family of transmembrane proteoglycans) to signaling pathways [80]. Syntenin-1 is well characterized and is known to regulate intracellular signaling pathways. In addition to its organizational and remodeling role in ECM organization and cell adhesion, it is also known to contribute to processes such as vesicle trafficking, exocytosis, and endocytosis [81]. In the context of sEV biogenesis, Syntenin-1 interacts with syndecans and ALIX (Programmed Cell Death 6 Interacting Protein) via its LYPX(n)L motif [81]. This interaction facilitates the connection of the syndecan–syntenin complex with ESCRT-I (Endosomal Sorting Complex Required For Transport I), which is essential for forming intraluminal vesicles (ILVs) within multivesicular bodies (MVBs) [82,83]. The entire process leads to the release of sEVs from cells. Studies report that *SDCBP* knockdown reduces the quantity of secreted sEVs and decreases the expression of key surface markers such as *ALIX* and *HSP70* (Heat Shock Protein 70 (Hsp70) Family Protein) [81,84]. Although less is known about Syntenin-2, which showed increased expression in the studied tissue sample, it can be hypothesized that its function is similar to Syntenin-1. Both proteins contain PDZ (a module that binds short peptide motifs at the extreme C-termini of other proteins) domains that enable interactions with various proteins, including cytoplasmic signaling molecules and transmembrane proteins [80]. There is evidence that the results for certain studied features are comparable for both proteins. For instance, both have been shown to interact with UNC93B1, a trafficking chaperone that regulates TLR signaling [80]. Their ability to associate with this chaperone suggests a potential role in the trafficking and sorting of TLR7 (Toll-like receptor 7) complexes into MVBs. TLR7 is predominantly expressed in immune cells [80], which are also present in the sulcular epithelium, suggesting that Syntenin-2 may similarly interact with these cell types.

CHMP4C is a member of the charged multivesicular protein (CHMP) family, which is part of ESCRT-III (Endosomal Sorting Complex Required For Transport III) [85,86]. It plays a role in membrane scission events, including cytokinesis, viral budding, and EV secretion, through its involvement in MVB formation [85,86]. Studies on CHMP4C have been primarily conducted in cancer-related contexts, and they indicate that its elevated expression increases sEV secretion [86,87]. For example, a study on pancreatic cancer cells and tissues showed that increased *CHMP4C* expression enhanced sEVs release, thereby facilitating cancer progression [87].

CHMP4C has also been investigated in the context of viral infections, as viruses utilize similar cellular machinery [85,88]. Both sEVs and enveloped viruses rely on the ESCRT complex for their formation [66]. Additionally, sEVs and viruses bud from the plasma membrane into the extracellular space following the prior involvement of MVBs [88]. Some studies have linked CHMP4C to both cancer and viral infections [86,88]. In a study on a cervical cancer cell line with HPV-induced oncogenic changes, elevated *CHMP4C* expression increased sEV secretion and metastasis, similar to findings in pancreatic cancer [86,87]. The role of CHMP4C has been confirmed not only in HPV infection but also in HSV-1, where it plays a key role in the final scission stage of membrane wrapping [89]. While these studies do not explicitly mention sEVs, they further support the strong involvement of CHMP4C in vesicle budding. This was demonstrated through depletion experiments, which led to a failure in the fission of endocytic tubules, preventing the virus from acquiring its final envelope and consequently reducing its production [89,90]. However, it is important to note that CHMP4C depletion has been observed in both HPV-1-infected and noninfected HeLa cells. In infected cells, the effects of this depletion were more pronounced, whereas in uninfected cells, they were less distinct and primarily affected the early stages of endosomal transport [89]. Further investigation of this phenomenon appears to be necessary. Regarding cancer, another study demonstrated that in p53-positive lung cancer cells, *CHMP4C* overexpression enhanced sEV production, whereas silencing the gene significantly reduced sEV secretion [91]. This suggests that in cells with high p53 levels, *CHMP4C* expression is also elevated [67]. Although this was again observed in cancer cells, it is essential to note that p53 is broadly associated with stress responses [91]. *CHMP4C* exhibited increased expression in the outer layers of the oral and sulcular epithelium, which are the first to come into contact with external factors. This may suggest that prolonged exposure to stress caused by mild irritation may drive *CHMP4C* expression and, consequently, increased sEV secretion [87]. To confirm, it would be necessary to assess levels of p53 in the studied section.

In cytokinesis, CHMP4C plays a role in membrane abscission, the final step in cell division that produces two daughter cells [92]. Given the high turnover rate of epithelial cells in the gingival epithelium, this may contribute to the observed elevated *CHMP4C* expression [86]. However, one study reported findings that differ from those in cancer cells. While Colombo et al. confirmed that CHMP4C regulates sEV secretion, as stated before, they found that in physiologically normal (non-cancerous, non-stressed) cells, increased sEV secretion resulted from *CHMP4C* silencing rather than its overexpression [93]. Although the studied gingival tissue sample was healthy, increased *CHMP4C* expression may not necessarily lead to reduced sEV secretion, as the study above would suggest. As stated before, constant exposure to pathogens, stress, and the high proliferative activity of epithelial cells may contribute to elevated *CHMP4C* expression and, consequently, enhanced sEV secretion.

VPS37B (Vacuolar Protein Sorting 37B) is a component of the ESCRT-I complex, which is involved in membrane remodeling, intracellular trafficking, and, indirectly, sEV biogenesis through its role in MVB formation and the sorting of ubiquitinated transmembrane proteins into the internal vesicles of multivesicular bodies [94,95]. Although the literature on its role in sEV biogenesis is limited, it provides interesting insights. Like *CHMP4C*, *VPS37B* has been studied in the context of viral infections.

HIV-1 Gag is a structural protein that plays a key role in viral assembly, budding, and release by accumulating on the inner surface of the plasma membrane [95]. This process partially mimics natural sEV release by hijacking the ESCRT system, making it useful for studying vesicle formation and secretion. In one study, researchers expressed HIV-1 Gag in HEK293T cells (human embryonic kidney 293T cells) using plasmid transfection [95]. Further analyses revealed that *VPS37B* and *VPS37C* knockdown dramatically inhibited a specific type of viral budding, whereas tethering VPS37B and VPS37C to HIV-1 Gag fully restored vesicle release [95]. Given that this virus hijacks the ESCRT system, which is also involved in sEV biogenesis, VPS37B is likely to have a similar role in sEV secretion.

Other studies have shown that *VPS37B* silencing in DLD1 cells, a human colorectal cancer cell line, destabilizes the entire ESCRT-I complex, thereby disrupting endosomal trafficking [96]. Additionally, ESCRT-I interacts with ALIX, a key protein in sEV biogenesis. It has been confirmed that VPS37B enhances the ESCRT-I/ALIX interaction, further suggesting its role in sEV production and release [95]. Moreover, VPS37B has been reported to colocalize with the sEV marker CD63, providing additional evidence for its involvement in sEV-related processes [94].

Some commonalities can be observed by analyzing the above-mentioned genes, most of which exhibit highly specific expression patterns associated with cluster 4. In addition to their documented or potential roles in sEV biogenesis, either ESCRT-dependent or -independent, they are also involved in membrane trafficking, stress responses, and immune signaling. Unlike the other genes, *MUC1* does not require ESCRT for secretion but instead utilizes RAB27A, particularly in response to bacterial exposure. SDCBP2 and VPS37B engage the ESCRT-I complex: SDCBP2 connects syndecans to ALIX, influencing MVB maturation and sEV release, while VPS37B is essential for endosomal sorting and vesicular transport. CHMP4C, in contrast, functions through ESCRT-III, facilitating sEV secretion by regulating membrane scission.

Although these genes operate through distinct mechanisms, each appears to contribute to sEV production, particularly in response to environmental stress. Their elevated expression in a specific tissue may indicate increased sEV production, likely driven by continuous exposure to pathogens and other stressors. This aligns with the localization of the sulcular epithelium, which, being in close proximity to the tooth, is constantly exposed to oral microbiota and serves as the primary soft tissue interface during daily oral hygiene practices.

While the primary focus of this study is on clusters 4 and 6, identified as the most associated with sEVs-related processes, it is also important to consider clusters covering anatomically similar epithelial areas. Clusters 2 and 3 correspond to the superficial and deeper layers of the oral epithelium. The literature indicates that it shares many similarities with the sulcular epithelium, such as cytokeratin expression patterns, which may be linked to similar responses to stress [97].

Additional clusters, namely 0, 1, 5, and 7, are also identified, and, as mentioned in the results, certain genes of interest exhibit noticeably higher expression in these clusters. These clusters, however, demonstrate lower heterogeneity, as illustrated in Figure 1C. As the elevated expression observed in these clusters is confined to individual cells or small subpopulations, it is, therefore, difficult to assess the potential of the entire cluster. As a result, clusters 0, 1, 5, and 7 are not analyzed in more detail in this study.

The remaining genes that exhibit specific expression patterns within epithelium-related clusters beyond clusters 4 and 6 include *CD9*, *CD63*, *CD81*, *FLOT1*, *RAB5A*, *RAB22A*, *RAB27B*, and *ANXA2*. Although *ANXA2* has also been referenced in the literature as an sEV marker, the other seven genes have been highlighted more often and h studied more broadly.

CD9, CD63, and CD81 belong to the family of membrane proteins known as tetraspanins and are integral components of the membranes of sEVs [98]. All three contribute to the formation of tetraspanin-enriched microdomains (TEMs), essential for sorting cargo into ILVs during the biogenesis of intracellular vesicles [98]. CD9 and CD63 influence the release of sEVs due to their involvement in membrane functions [99]. CD9 also plays a role in cell adhesion by stabilizing intercellular connections, thereby affecting cell-to-cell communication [100,101]. CD81 is additionally involved in the regulation of cellular signaling, particularly in transmitting signals associated with immune responses [102,103]. CD63 is closely linked to MVBs and late endosomes [104,105]. It plays a key role in cargo maturation and sorting and contributes to the removal of damaged proteins through lysosomal degradation [104,106]. Together, these tetraspanins are key regulators of sEVs release and intracellular cargo management, underlining their importance in maintaining cellular communication and homeostasis [98,99,100,102,104,107]. Additionally, FLOT1 is a protein associated with lipid rafts and is involved in clathrin-independent endocytosis [108]. According to the literature, it plays a crucial role in the biogenesis of sEVs, particularly in the organization of lipid microdomains in endosomal membranes [109,110]. This process is fundamental for protein sorting during sEV biogenesis.

RAB5A, RAB22A, and RAB27B are members of the Rab family of small GTPases that regulate vesicular transport [111]. RAB5 is pivotal in early endocytosis and endosome maturation. It influences the availability of structures (soon to be cargo) to be packaged into sEVs [112]. RAB22A is involved in early endosome and multivesicular body (MVB) structures, containing LC3 (Light chain 3) and calnexin, which are later involved in sEV biogenesis [52,113]. RAB27B operates in later stages, specifically the docking and fusion of MVBs with the plasma membrane [53]. This step is critical for releasing sEVs and their cargo into the extracellular space. In addition to their vesicle-related functions, RAB5A supports immune response regulation by reacting to exposition to pathogens [114], such as resident or transient bacteria near the gingiva. RAB22A contributes to the regulation of autophagy, which aids in cellular recycling and homeostasis [115], and RAB27B participates in repair processes by facilitating the transport of growth factors and enzymes involved in tissue regeneration, as well as in angiogenesis, promoting the formation of new blood vessels [116]. As can be seen, these proteins play a key role in the biogenesis and release of sEVs while also engaging in immune responses, tissue repair, and homeostasis in gingival tissues.

The final gene, *ANXA2*, plays a role in the immune response by regulating cytokine production and participating in the migration of fibroblasts and keratinocytes [117,118,119]. The ANXA2 protein binds lipids in a calcium-dependent manner, making it a general regulator and a phospholipid-binding protein that plays a critical role in membrane dynamics and cytoskeletal organization [120]. In the context of sEV biogenesis, *ANXA2*, like many of the previously mentioned genes, contributes to the fusion of MVBs with the plasma membrane, enabling the release of vesicles into the extracellular environment [121].

Although the genes associated with the epithelium-related clusters collected in Figure 5 exhibit the highest expression in cluster 2, it is important to note that the expression levels of these genes are not that different in other epithelium-related clusters. All these genes, particularly those critical for sEV biogenesis, such as the tetraspanins (CD9, CD63, CD81), undoubtedly contribute valuable insights into the processes of sEV biogenesis in gingival tissue. However, in the case of genes such as *CD9*, *CD63*, *CD81*, *FLOT1*, *RAB5A*, *RAB22A*, *RAB27B*, and *ANXA2*, their expression patterns do not clearly establish them as specific markers of sEV biogenesis in the identified clusters of this particular tissue section.

With the use of spatial transcriptomics, this study analyzed gene expression patterns associated with sEV biogenesis across different cell clusters in a healthy human gingival tissue section, with a particular focus on clusters encompassing the superficial and deeper regions of the sulcular epithelium. The expression profiles of MUC1, SDCBP2, and VPS37B suggest that this region plays a role in barrier function, immune response, and microbial interactions, likely influenced by the constant exposure of the sulcular epithelium to the oral microbiota. Other genes, including CD9, CD63, CD81, FLOT1, and various members of the RAB family, were also identified in the epithelial clusters; however, despite being well-established sEV markers, their expression is not most pronounced in the sulcular epithelium, the region identified through GO analysis as exhibiting the highest biological activity related to sEVs. The remaining clusters display high heterogeneity, challenging detailed analysis and necessitating further investigation. Overall, the findings highlight the potential role of the sulcular epithelium, both superficial and deeper layers, in intensive sEV biogenesis, likely as an adaptive response to environmental stress and prolonged microbial exposure due to its anatomical location in the oral cavity.

## 5. Conclusions

Considering current trends in medical development and the growing demand for expanding knowledge in specific areas, research on regenerative processes is expected to increase and progress significantly. While attention is often drawn to the grand outcomes of such studies, the underlying mechanisms leading to these results are equally important.

In this meta-analysis, we integrate current knowledge on the oral mucosa—one of the most rapidly regenerating tissues in the human body—and small extracellular vesicles, which have a well-established role in regenerative processes, by analyzing available transcriptomic data in this context. Our study suggests that the sulcular epithelium of the gingiva exhibits significant regenerative potential due to its potential association with the biogenesis of small extracellular vesicles. Additionally, we identify potential markers of sEV biogenesis that have not been extensively studied in this context and confirm the presence of well-established sEV markers.

We acknowledge the limitations of our study, particularly the absence of functional assays or the direct experimental validation of extracellular vesicle production. Nevertheless, our computational and descriptive approach provides insights and observations that can guide future laboratory research in this field, including spatial transcriptomics. These data may contribute to a deeper understanding of the regenerative potential of gingival tissue and provide new insights into these processes in other regions of the oral mucosa. Furthermore, our observations may assist research groups studying regeneration by identifying a specific area of the gingiva that warrants particular attention in terms of sEV biogenesis.

While we recognize that cell-to-cell and tissue-to-tissue communication is essential for obtaining a comprehensive understanding, a “bigger picture”, precisely selecting the regions of investigation allowed us to achieve targeted results in the areas of interest, ultimately providing a “molecular-sized picture” with the highest resolution.

## Figures and Tables

**Table 1:** Selected genes with a potential or documented role in sEV biogenesis.

	Source	Gene Name			Citation
1	Lipid Raft Endocytosis and Exosomal Transport Facilitate Extracellular Trafficking of Annexin A2, Valapala et al.		*ANXA2*		[47]
2	Incorporating extracellular vesicle markers varies among vesicles with distinct surface charges, Maeda et al.	*CD63*	*CD81*	*CD9*	[48]
			*FLOT1*		
3	Silencing of human papillomavirus (HPV) E6/E7 oncogene expression affects both the contents and the amounts of extracellular microvesicles released from HPV-positive cancer cells, Honegger et al.		*CHMP4C*		[49]
4	MUC1-C is a master regulator of MICA/B NKG2D ligand and exosome secretion in human cancer cells, Morimoto et al.		*MUC1 (MUC1-C)*		[50]
5	RAB5A is associated with genes involved in exosome secretion: Integration of bioinformatics analysis and experimental validation, Gorji-bahri et al.		*RAB5A*		[51]
6	RAB22A as a predictor of exosome secretion in the progression and relapse of multiple myeloma, Fan et al.		*RAB22A*		[52]
7	Rab27a and Rab27b control different steps of the exosome secretion pathway, Ostrowski et al.		*RAB27B*		[53]
8	MDA-9/Syntenin: An emerging global molecular target regulating cancer invasion and metastasis, Das et al.		*SDCBP2*		[54]
9	Embryonic signals mediate extracellular vesicle biogenesis and trafficking at the embryo–maternal interface, Guzewska et al.		*VPS37B*		[55]

## Data Availability

Data are contained within the article.

## Abbreviations

The following abbreviations are used in this manuscript:

*A2M*	Alpha-2-Macroglobulin
AG	Attached gingiva
*ALIX*	Programmed Cell Death 6 Interacting Protein
*ANGPTL7*	Angiopoietin Like 7
*ANXA*, *2*, *5*, *11*	Annexin, II, V, XI
*ATP6V0A4*	ATPase H+-Transporting V0 Subunit A4
*AQP5*	Aquaporin 5
*BAG6*	BAG Cochaperone 6
*CCNJL*	Cyclin J-like
*CCL14*	Chemokine C-C motif Ligand 14
*CCR2*	C-C Motif Chemokine Receptor 2
*CD247*, *63*, *73*, *81*, *82*, *9*	CD247, 63, 73, 81, 82, 9 molecule
*CERK*	Ceramide Kinase
*CHMP1A*, *2A*, *4B*, *4C*	Charged Multivesicular Body Protein 1A, 2A, 4B, 4C
*CLCA4*	Chloride Channel Accessory 4
*CLTC*	Clathrin Heavy Chain
*COL11A1*	Collagen Type XI Alpha 1 Chain
CRC	Colorectal cancer
*CYP24A1*	Cytochrome P450 Family 24 Subfamily A Member 1
DEG	Differentially expressed gene
*DUOXA2*	Dual Oxidase Maturation Factor 2
ECM	Extracellular matrix
EGFR	Epidermal Growth Factor Receptor
*ESCRT-I*, *-III*	Endosomal Sorting Complex Required For Transport I, III
EVs	Extracellular vesicles
FG	Free gingiva
*FGF22*	Fibroblast Growth Factor 22
*FLOT1*	Flotillin-1
*FUT6*	Fucosyltransferase 6
GEO	Gene Expression Omnibus
GG	Gingival groove
GMSC	Gingival mesenchymal stem cell
GO	Gene Ontology
*GZMA*	Granzyme A
H_2_O_2_	Hydrogen peroxide
H&E	Hematoxylin and eosin
*HSP70*	Heat Shock Protein 70 (Hsp70) Family Protein
*HSP90AA1*	Heat Shock Protein 90 Alpha Family Class A Member 1
*HSPA8*	Heat Shock Protein Family A (Hsp70) Member 8
*ICAM1*	Intercellular Adhesion Molecule 1
*IGHM*	Immunoglobulin Heavy Constant Mu
*IGHA1*	Immunoglobulin Heavy Constant Alpha 1
*IGHG2*	Immunoglobulin Heavy Constant Gamma 2
*IGHV3-30*	Immunoglobulin Heavy Variable 3-30
*IGHV6-1*	Immunoglobulin Heavy Variable 6-1
*IGKV*	Immunoglobulin Kappa Variable Cluster
ILV	Intraluminal vesicle
*ITGB3*	Integrin Subunit Beta 3
JE	Junctional epithelium
*LAMC2*	Oxidative Stress-Induced Growth Inhibitor 1
LC3	Light chain 3
*LCE2A*, *2B*, *6A*	Late Cornified Envelope 2A, 2B, 6A
*LGALS3BP*	Galectin 3 Binding Protein
LP	Lamina propria
MAPK/ERK	Mitogen-Activated Protein Kinase/Extracellular Signal-Regulated Kinase
*MFGE8*	Milk Fat Globule EGF and Factor V/VIII Domain Containing
miRNA	Microribonucleic Acid
MISEV	Minimal Information for Studies of Extracellular Vesicles
mRNA	Messenger Ribonucleic Acid
*MS4A1*	Membrane Spanning 4-Domains A1
*MUC1*	Mucin 1
MVB	Multivesicular body
*MZB1*	Marginal Zone B and B1 Cell-Specific Protein 1
NK (cells)	Natural killer
OE	Oral epithelium
*OSGIN1*	Oxidative Stress-Induced Growth Inhibitor 1
PCA	Principal component analysis
*PEG3*	Paternally Expressed 3
*PLA2G2F*	Phospholipase A2 Group IIF
*PLD2*	Phospholipase D2
*PTEN*	Phosphatase and Tensin Homolog
*RAB11A*, *B*	RAB11A, B, Member RAS Oncogene Family
*RAB22A*	RAB22A, Member RAS Oncogene Family
*RAB27A*, *B*	RAB27A, B, Member RAS Oncogene Family
*RAB35*	RAB35, Member RAS Oncogene Family
*RAB5A*	RAB5A, Member RAS Oncogene Family
*RAB7A*	RAB7A, Member RAS Oncogene Family
RNA	Ribonucleic Acid
SE	Sulcular epithelium
*SDCBP2*	Syndecan Binding Protein 2
sEVs	Small extracellular vesicles
*SMPD3*	Sphingomyelin Phosphodiesterase 3
*SNAP23*	Synaptosome-Associated Protein 23
SNN	Shared nearest neighbor
*SPIB*	Spi-B Transcription Factor
*STX4*	Syntaxin 4
T	Tooth
TLR7	Toll-like receptor 7
*TNFRSF9*	TNF Receptor Superfamily Member 9
*TP53*	Tumor Protein P53
*TRAJ20*	Cytochrome P450 Family 24 Subfamily A Member 1
*TSG101*	Tumor Susceptibility 101
UMAP	Uniform Manifold Approximation and Projection
UNC93B1	Unc-93 Homolog B1, TLR Signaling Regulator
*VAMP7*	Vesicle-Associated Membrane Protein 7
*VPS37B*, *C*	VPS37B, C Subunit Of ESCRT-I
*VPS4A*, *B*	Vacuolar Protein Sorting 4 Homolog A, B

## Appendix A

**Table A1:** Genes described in the literature as potential or confirmed sEV markers.

	Source	Gene Name			Citation
1	The incorporation of extracellular vesicle markers varies among vesicles with distinct surface charges, Maeda et al.	*CD63*	*CD81*	*CD9*	[48]
			*FLOT1*		
2	Identification of specific markers for human pluripotent stem cell-derived small extracellular vesicles, Chen et al.	*OCT4*	*PODXL*	*OCT4*	[122]
3	Pentapartite fractionation of particles in oral fluids by differential centrifugation, Hiraga et al.	*AQP5*	*CD133*	*CD63*	[123]
		*CD81*		*CD9*	
4	ExoCarta: Exosome markers, Mathivanan et al.	*A2M*	*ACLY*	*ACTB*	[124]
		*ACTG1*	*ACTN4*	*AHCY*	
		*ALB*	*ALDOA*	*ANXA1*	
		*ANXA11*	*ANXA2*	*ANXA4*	
		*ANXA5*	*ANXA6*	*ARF1*	
		*ATP1A1*	*BSG*	*CCT2*	
		*CCT3*	*CCT5*	*CD63*	
		*CD81*	*CDC42*	*CFL1*	
		*CLIC1*	*CLTC*	*EEF1A1*	
		*EEF2*	*EHD4*	*ENO1*	
		*EZR*	*FASN*	*FLNA*	
		*FLOT1*	*FN1*	*GAPDH*	
		*GDI2*	*GNAI2*	*GNAS*	
		*GNB1*	*GNB2*	*GSN*	
		*HIST1H4A*	*HIST1H4B*	*HIST2H4A*	
		*HSP90AA1*	*HSP90AB1*	*HSPA1A*	
		*HSPA5*	*HSPA8*	*ITGA6*	
		*ITGB1*	*KPNB1*	*LAMP2*	
		*LDHA*	*LDHB*	*LGALS3BP*	
		*MFGE8*	*MSN*	*PDCD6IP*	
		*PFN1*	*PGK1*	*PKM*	
		*PPIA*	*PRDX1*	*PRDX2*	
		*PTGFRN*	*PTGFRN*	*RAB14*	
		*RAB1A*	*RAB5A*	*RAB5B*	
		*RAB5C*	*RAB7A*	*RAB8A*	
		*RAC1*	*RAN*	*RAP1B*	
		*RHOA*	*SDCBP*	*SLC16A1*	
		*SLC3A2*	*STOM*	*TCP1*	
		*TFRC*	*THBS1*	*TKT*	
		*TPI1*	*TSG101*	*TUBA1A*	
		*TUBA1B*	*TUBA1C*	*UBA1*	
		*VCP*	*YWHAB*	*YWHAE*	
		*YWHAG*	*YWHAH*	*YWHAQ*	
			*YWHAZ*		
5	Differential Expression of Keratinocyte-Derived Extracellular Vesicle Mirnas Discriminate Exosomes From Apoptotic Bodies and Microvesicles, Than et al.	*HSP70*	*AGO2*	*TSG101*	[125]
		*CD9*	*CD163*	*CD9*	
6	Advances in mesenchymal stem cell exosomes: a review, Tang et al.	*CD10*	*CD133*	*CD29*	[126]
		*CD44*	*CD63*	*CD73*	
		*CD73*	*CD105*	*FLOT1*	
		*ICAM1*	*ALIX*	*EPCAM*	
		*TSG101*	*CD81*	*CD106*	
7	Endosomal signalling via exosome surface TGFβ-1, Shelke et al.		*TGFB1*		[127]
8	Neutral sphingomyelinase 2 controls exosome secretion by counteracting V-ATPase-mediated endosome acidification, Choezom et al.	*ATP6V1A*		*ATP6VE1*	[128]
9	ITGB3-mediated uptake of small extracellular vesicles facilitates intercellular communication in breast cancer cells, Fuentes et al.		*ITGB3*		[129]
10	Exocyst controls exosome biogenesis via Rab11a, Bai et al.		*RAB11A*		[130]
11	Exosome-dependent immune surveillance at the metastatic niche requires BAG6 and CBP/p300-dependent acetylation of p53, Schuldner et al.		*BAG6*		[131]
12	Role of Ceramides and Lysosomes in Extracellular Vesicle Biogenesis, Cargo Sorting and Release, Horbay et al.		*CERK*		[132]
13	Phospholipase D and phosphatidic acid in the biogenesis and cargo loading of extracellular vesicles, Egea-Jimenez et al.		*PLD2*		[133]
14	PTEN Deficiency Facilitates Exosome Secretion and Metastasis in Cholangiocarcinoma by Impairing TFEB-mediated Lysosome Biogenesis, Jiang et al.		*PTEN*		[134]
15	Regulation of cargo selection in exosome biogenesis and its biomedical applications in cancer, Lee et al.		*CD8*		[135]
16	Exosomal CCL2 from Tubular Epithelial Cells Is Critical for Albumin-Induced Tubulointerstitial Inflammation, Lv et al.		*IL6*		[136]
17	MUC1- C is a master regulator of MICA/B NKG2D ligand and exosome secretion in human cancer cells, Morimoto et al.		*MUC1 (MUC1-C)*		[50]
18	Embryonic signals mediate extracellular vesicle biogenesis and trafficking at the embryo–maternal interface, Guzewska et al.		*VPS37B*		[55]
19	MDA-9/Syntenin: An emerging global molecular target regulating cancer invasion and metastasis, Das et al.		*SDCBP2*		[54]
20	The ESCRT-III Protein CHMP1A Mediates Secretion of Sonic Hedgehog on a Distinctive Subtype of Extracellular Vesicles, Coulter et al.		*CHMP1A*		[137]
21	Identification of the SNARE complex that mediates the fusion of multivesicular bodies with the plasma membrane in exosome secretion, Liu et al.		*SNAP23*, *VAMP7*		[138]
22	*SMPD3*-mediated extracellular vesicle biogenesis inhibits oligodendroglioma growth, Balakrishnan et al.		*SMPD3*		[139]
23	VAMP5 and distinct sets of cognate Q-SNAREs mediate exosome release, Matsui et al.		*STX4*		[140]
24	ALIX- and ESCRT-III–dependent sorting of tetraspanins to exosomes, Larios et al.		*CHMP4B*		[141]
25	Regulation of exosome secretion by Rab35 and its GTPase-activating proteins TBC1D10A–C, Hsu et al.		*RAB35*		[142]
26	Vps4A-mediated tumor suppression upon exosome modulation? Akrap et al.		*VPS4A*		[143]
27	p-AKT/VPS4B regulates the small extracellular vesicle size in venous malformation endothelial cells, Lai et al.		*VPS4B*		[144]
28	A novel TP53 pathway influences the HGS-mediated exosome formation in colorectal cancer, Sun et al.		*TP53*		[145]
29	Silencing of human papillomavirus (HPV) E6/E7 oncogene expression affects both the contents and the amounts of extracellular microvesicles released from HPV-positive cancer cells, Honegger et al.		*CHMP4C*		[49]
30	LRRK2 secretion in exosomes is regulated by 14-3-3, Fraser et al.		*LRRK2*		[146]
31	Rab27a and Rab27b control different steps of the exosome secretion pathway, Ostrowski et al.		*RAB27A*, *RAB27B*		[53]
